# Synthesis and Thermal Degradation Studies of Melamine Formaldehyde Resins

**DOI:** 10.1155/2014/940502

**Published:** 2014-11-09

**Authors:** Sami Ullah, M. A. Bustam, M. Nadeem, M. Y. Naz, W. L. Tan, A. M. Shariff

**Affiliations:** ^1^Research Center for CO_2_ Capture, Department of Chemical Engineering, Universiti Teknologi PETRONAS, 31750 Tronoh, Perak, Malaysia; ^2^Petroleum Development Oman LLC, 100 Muscat, Oman; ^3^Department of Fundamental and Applied Science, Universiti Teknologi PETRONAS, 31750 Tronoh, Perak, Malaysia

## Abstract

Melamine formaldehyde (MF) resins have been synthesized at different reaction temperature and pH values. Different molar ratios of melamine and formaldehyde were used to synthesize the corresponding resins. The prepared resin samples were characterized by using molecular weight determination viscometry and thermogravimetric analysis (TGA). The maximum percentage of solid content (69.7%) was obtained at pH 8.5 and 75°C temperature. The molecular weight of MF resin was increased with an increase of melamine monomer concentration. The highest residual weight 14.125 wt.% was obtained with sample 10.

## 1. Introduction 

Resin is a polymeric material which is used for impregnating and bonding layers of laminate flooring. MF resin is a thermosetting resin that changes irreversibly under the influence of heat from a fusible and soluble material into a one which is infusible and insoluble through the formation of covalently cross-linked and thermally stable network [1]. Adhesives and the physicochemical phenomenon of adhesion play an important role in more than 70% of all wood-based materials in use today [2, 3]. This includes not only bonded wood products but also paper products, paints, and finishes. Natural adhesives that have been used by the forest products industry include adhesives derived from starch, soybeans, animal waste, and byproducts of the meat processing adhesives derived from starch, soybeans, animal waste and byproducts of the meat processing, tanning industries, casein from skim milk, and so forth [4–6]. These protein-based adhesives (soy, blood, and casein) were most commonly used materials over the years. However, adhesives derived from natural sources were limited to dry interior applications [7]. Efficiency in converting trees and waste wood to useful products will help to ensure the continual conservation of forest resources [8, 9].

Cross-linkable polymers are generally referred to as thermosets, or thermosetting resins [10, 11]. From a processing point of view, the above distinction of thermoplastic and thermoset implies that the former can, in principle, be extruded and molded many times, while the latter can be processed at high temperature only once before cross-linking reactions render the polymer hard and solid. These polymers, upon cure, are insoluble and do not soften on heating [12, 13]. Amino resins, phenolic resins, and isocyanates are the three most important thermosetting resin systems used by the wood products industry [14]. Other thermosetting types include the epoxy resin, the unsaturated polyester resin, urethane foams, the alkyds widely used for surface coating, and minor types [15]. A drawback of these materials is the potential release of formaldehyde during and after cure and poor weather ability. Melamine mouldings have somewhat better weather ability than the urea mouldings [16, 17].

Melamine formaldehyde resins were discovered in Germany in the early thirties but there was no commercial development in this century. The term amino plastics has been coined to cover a range of resinous polymers produced by interaction of amines or amides with aldehydes [18, 19]. In many respects, the chemistry of the formation of melamine-based resins is very similar to that of UF resins. However, the addition of formaldehyde to the amino groups of melamine is faster and more complete than is the addition of formaldehyde to urea [20]. Thus, complete hydroxymethylation of melamine occurs [21], which, as mentioned above, is not the case with urea. Another important difference is that the condensation reactions of the hydroxymethylated melamine occur not only under acid conditions but under neutral and slightly alkaline conditions as well [22]. In addition, products bonded with MF resins seem to be less susceptible to the release of formaldehyde than are products bonded with UF resin. The surface coating materials improved the physical and mechanical properties of particle board [23].

The objective of this work is synthesis and characterization of MF resins at different molar ratios of melamine and formaldehyde and determination of the morphology, molecular weight, and solid content of synthesized resin samples. The prepared resin samples were characterized by using molecular weight determination viscometry, field emission scanning electron microscopy (FESEM), and thermogravimetric analysis (TGA).

## 2. Materials and Methods

Melamine was used as received from Fluka. Formaldehyde (37 wt% in water), NaOH, and boric acid were used as received from Merck. The steps involved in synthesis and characterization of the targeted resins have been detailed in next sections.

### 2.1. Synthesis of MF Resin

A similar method was followed for synthesis of MF resins as explained by Binder and Dunky [1], Fink [24], and Jahromi [25]. Polymerization of melamine and formaldehyde at different molar ratios was carried out in demineralized water of 0.06 siemens conductivity at 75°C. The different molar quantities of melamine ranging from 0.1 M to 0.25 M and fixed formaldehyde of 1 M were used at constant temperature of 75°C and pH 8.5. The polymerization reaction was conducted for two hours under constant agitation for obtaining higher degree of conversion. The pH of the reaction mixture was maintained at 8.5 by use of NaOH. The final product was obtained in the form of clear, viscous, and transparent liquid. The end point of the reaction was checked regularly by water tolerance method. Similar procedure was repeated again and ten samples of melamine resin were synthesized with different monomer ratios of melamine and formaldehyde. The synthesized MF resin samples were characterized further and manipulated in Results and Discussion section.

### 2.2. Molecular Weight Determination of MF Resin

Solid contents of the resin samples were determined by heating them in an oven. 5 g of each sample was taken on an aluminum pan and placed in an oven at 125°C for four hours. The molecular weights of polymer resin samples were determined by using the viscometry technique. A general procedure was adopted for the determination of molecular weight. Flow time of each dilution of polymer solution was noted using Cannon-Ubbelohde viscometer. The experiments were carried out at constant temperature of 27°C ± 0.1°C. Flow time for the solvent (water) was noted by taking the measured volume of solvent in the viscometer. Three concordant readings of flow time were noted and averaged for achieving the maximum accuracy in the results. The viscometer was then emptied and dried and 10 mL of polymer solution of 8 g/10 mL of water concentration was taken. Again three concordant readings of flow time were taken. The solution was then diluted to 7 mg/10 mL by adding 1.42 mL of the solvent in the same solution and the corresponding flow time was noted again. Similarly, 6 mg/10 mL, 5 mg/10 mL, and 4 mg/10 mL dilutions were made by adding 1.91, 2.67, and 4 mL of solvent, respectively. All solutions were made by staking 8 mg of polymer on the basis of solid content. The corresponding viscosity average molecular weight was measured. In order to obtain better results, the Maroon and Razink equation was used to determine the viscosity average molecular weight:

(1) $$\begin{array}{c} \frac{\Delta }{c^{2}}=\frac{1}{2}{\left[\eta \right]}^{2}+\left(K_{1}-\frac{1}{3}\right){\left[\eta \right]}^{3}C, \end{array}$$

where Δ = *η*_sp−ln⁡_ *η*_*r*_. The values of Δ/*c* ^2^ were plotted versus *C* and straight line interception was equal to 1/2 [*η*]^2^.

Efflux time of the pure solvent is given by *t*_*o*_. The relative viscosity is the viscosity of the polymer solutions to the viscosity of the pure solvent. This is done by taking the efflux time of the polymer solution at a given concentration (we call this *t*) and dividing it by “*t*_*o*_” as explained below:

(2) $$\begin{array}{c} \frac{\text{Efflux}\;\text{time}\;\text{of}\;\text{solution}}{\text{Efflux}\;\text{time}\;\text{of}\;\text{solvent}}=\text{Relative}\;\text{viscosity},\\ \frac{t}{t_{o}}=\eta_{t}. \end{array}$$

The specific viscosity was measured by taking the difference in the efflux times of the solution and the pure solvent. In other words, the efflux time of the pure solvent “*t*_*o*_” was subtracted from the efflux time of the solution “*t*” as explained below:

(3) Efflux  time  of  solution−Efflux  time  of  solventEfflux  time  of  solvent =Specifice  viscosity,t−toto=ηsp.

The intrinsic viscosity was measured by using the following equation (4):

(4) $$\begin{array}{c} \left[\eta \right]=\frac{\eta_{\text{sp}}}{c}. \end{array}$$

From the flow time relative velocity (*η*_*r*_), specific viscosity (*η*_sp_) and ultimately the intrinsic viscosity ([*η*]) were calculated for the synthesized samples. Herein, the molecular weight of the polymers was also determined by using Mark Houwink equation Allcock and Lampe [26]:

(5) $$\begin{array}{c} \left[\eta \right]=K{\left[M\right]}^{a}, \end{array}$$

where [*η*] is intrinsic viscosity, “*M*” is the molecular mass of the polymer, “*K*” represents the characteristics of the polymer and solvent, and “*a*” is a constant and a function of the shape of the polymer coil in the solution. In case of melamine formaldehyde resin the value of “*a*” is usually 0.6 and “*K*” is 0.076. In this experiment, the values of “*K*” and “*a*” were based on the behavior of homopolymers in the aqueous phase.The values of these constants depend upon the polymer solvent interactions. 

*Thermogravimetric Analysis (TGA)*. The thermogravimetric analyses of synthesized sample (approximately 10 mg) were carried out over the whole range of temperature (50–700°C) with ramping rate of 10°C/min. In present case, TGA Q50 PerkinElmer was serving this purpose. This characterization was performed to determine the residual weight of MF resin.

*Differential Thermal Analysis (DTA)*. The DTA analysis of intumescent coating (approx. 10 mg) was carried out at a heating rate of 10°C/min in nitrogen gas, set at the flow rate of 20 mL/min over the whole temperature range of 50 to 800°C. The data was recorded using Pyris Player data analyzer.

## 3. Results and Discussion

The main use of melamine is as a reactive intermediate for the manufacturing of MF resins. The reaction conditions including time, temperature, formaldehyde/melamine (F/M) ratio, pH, and catalyst influence the composition and structure of the resin that makes up the adhesive. The MF resin adhesive needs to be activated to give good polymerization to the final product. Similar to UF resin, this usually involves lowering the pH and raising the temperature. The catalysts added to the MF resin are either acids or acid precursors that liberate acid upon heating. Often hardener such as ammonium chloride or sulfate is added which generates hydrogen chloride or hydrogen sulfate plus ammonia (migrates from the adhesive). In most applications, the products are heat-cured. Although the bonded products show respectable water resistance, phenol-containing resins are preferred for exterior uses in the United States. Unlike the UF resin adhesives, MF resins do not show degradation during water boiling [22, 27]. They do show some loss of bond strength during acceleration and exterior exposure tests [28, 29].

**Figure 1:**
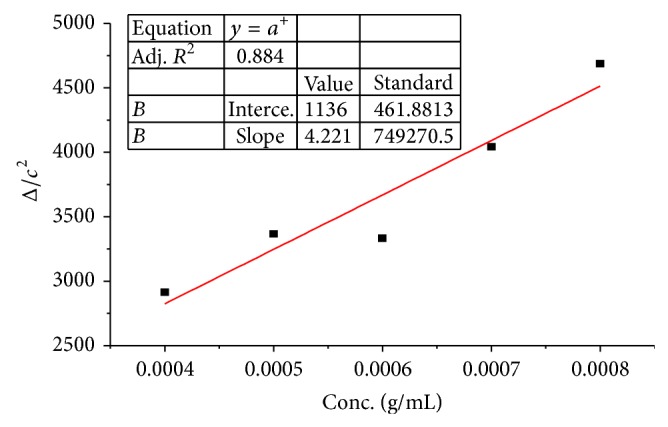
Dependence of Δ/*c* ^2^ on the concentration of sample 6 (*R* ^2^ = 91.4%, *R* ^2^(adj) = 88.5%).

The first step in MF curing is the addition of the formaldehyde to the melamine as shown in Figure 1  [22]. Melamine is a good nucleophile; the addition reaction with the electrophilic formaldehyde occurs under most pH conditions, although the reaction rate is slower at neutral pH. The melamine reacts with up to six formaldehyde groups to form two methylol groups on each exocyclic amine group. The mixture of hydroxyl methyl compounds then reacts by condensation to form the resin. The addition reaction is reversible, though generally the equilibrium is far to the right side. On the other hand, the condensation reaction to form oligomers and polymers is not very reversible which is important for the water resistance of the product and makes it different from UF resin. It is evident from the dimers illustration that many isomers can be produced. Considering that each melamine has three amine groups, with each amine group having up to two hydroxymethyl groups attached, formation of both methylene and bismethylene ether bridges occurs, and formation of dimers, trimers, and higher oligomers takes place. As a result, the reaction chemistry rapidly reaches its highest complexity. Pizzi and Mittal have already studied the chemistry of some of these reactions [22]. 

*Solid Content*. The solid content of the prepared samples was ranging from 40 to 69.7%. The extracted data has been summarized in Table 1. It was found that the solid contents were increased by increasing the molar concentration of melamine in the reaction carried out at temperature of 70°C and pH 8.5. The maximum amount of solid content 69.6% was obtained by using 0.22 M concentration of melamine and 3 M formaldehyde.

**Figure 2:**
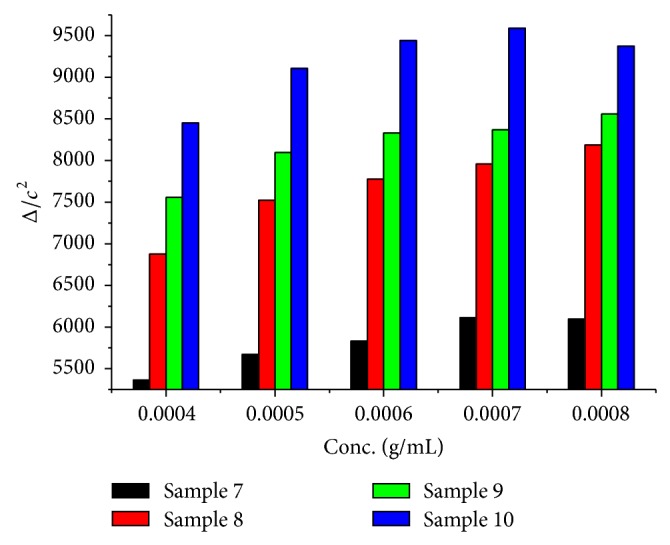
Dependence of Δ/*c* ^2^ on the concentration of sample 7–10.

**Figure 3:**
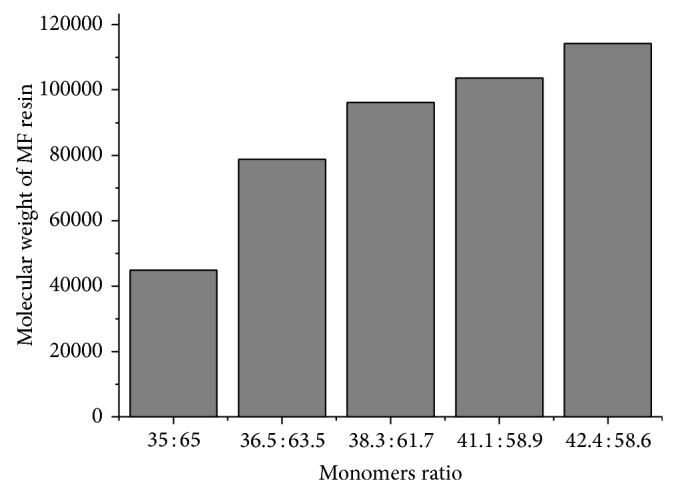
Molecular weight of MF resin samples (6–10) versus monomers ratios.

*Molecular Weight*. The molecular weight of five selected samples (having high solid content) was measured and presented in Table 1. The intrinsic viscosity of sample 6 was determined by extrapolating the graph between concentration and Δ/*c* ^2^. At zero concentration, this plot gives 1/2 [*η*]^2^ which was 2600. Thus intrinsic viscosity [*η*] was found to be 72.11. From intrinsic viscosity, the molecular weight was calculated by applying (1) and found to be 44860 as expressed in Table 2 and Figure 1. Similar procedure was repeated for samples 7, 8, 9, and 10 and the measured intrinsic viscosities were 117.47, 123.28, and 131.14, respectively. The corresponding values of Δ/*c* ^2^ have been provided in Table 3 and Figure 2. The molecular weights of the samples 7, 8, 9, and 10 were 78829, 96158, 103703, and 114218, respectively. The data in Table 4 and Figure 3 reveals that sample 10 has the highest molecular weight of 110046 followed by samples 9, 8, 7, and 6. The molar ratio of the monomers used in this experiment was 0.22 : 3, while keeping other components the same as used in other samples. From these results it was clear that molecular weight increases with an increase in melamine concentration in the polymer. MF sample 10 was having the maximum molecular weight and solid content.

**Figure 4:**
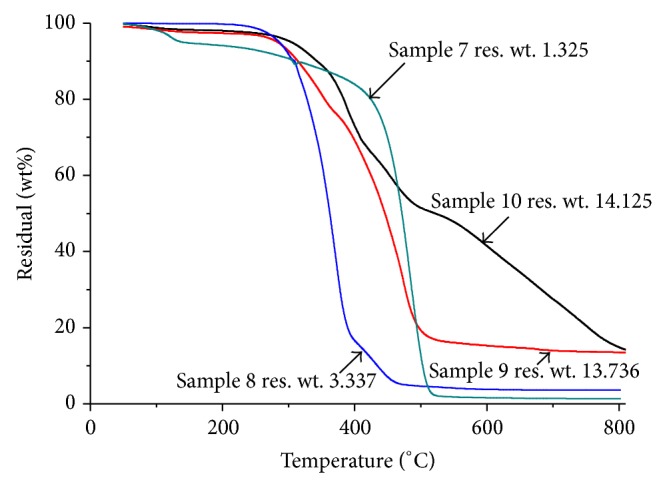
TGA of MF resins.

**Figure 5:**
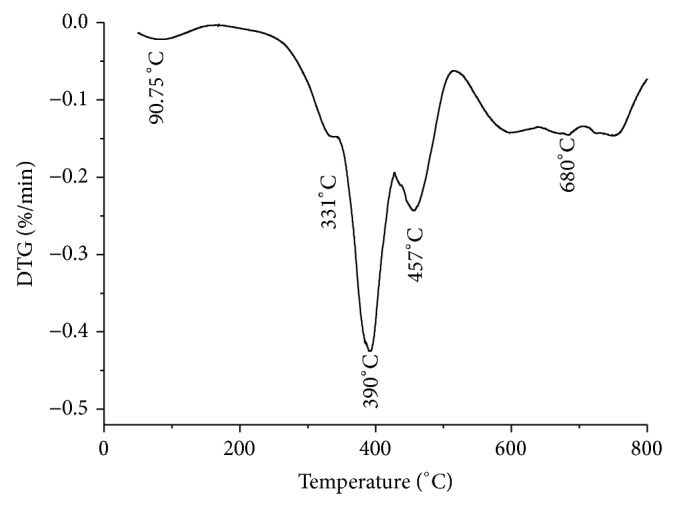
DTGA of sample 10.

*Residual Weight of MF Resin*. The weight losses observed during the heating program on the melamine formaldehyde resin have been presented in Figure 4. The residual weights of samples 7, 8, 9, and 10 have been 1.325, 3.337, 13.763, and 14.125% at 800°C, respectively, as shown in Figure 4. For each temperature range, the values of the partial weight losses and their characteristic temperatures determined from the minimum of the four peaks observed on the derived curve of sample 10 are reported in Figure 5. The major weight losses were observed in the temperature range of 300 to 450°C, which may correspond to the structural decomposition of the resins. The main four temperature ranges where weight losses appeared are 50 to 125°C, 125 to 335°C, 335 to 390°C, and 390 to 475°C and the last one for temperatures higher than 440°C. During the first weight loss (*T* = 90.75°C), the DTG analysis gave 4% weight loss due to water loss. Therefore, it may be concluded that this first weight loss is primarily due to the water vaporization. The second weight loss (*T* = 331°C) was due to formaldehyde, methanol, and amine.

The polycondensation reaction had taken place at temperatures above 331°C when a number of independent reactions involving both side-chain and ring degradation gave rise to the products. This means that some molecules of melamine can be sublimated at a temperature lower than the sublimation temperature generally observed at 345°C [30, 31]. During the third weight loss (*T* = 390°C), formaldehyde, methanol, amine, and NH_3_ were released. For the last weight losses (at temperature greater than 450°C), the results were consistent with what is generally observed for the thermal degradation of melamine [17]. Thus according to Ferra et al. [5], it is assumed that MF gradually forms cyameluric structures. Above 660°C, the MF resin condensate went through wide degradation with formation of volatile products including CO_2_, HCN, and CO [30].

**Figure 6:**
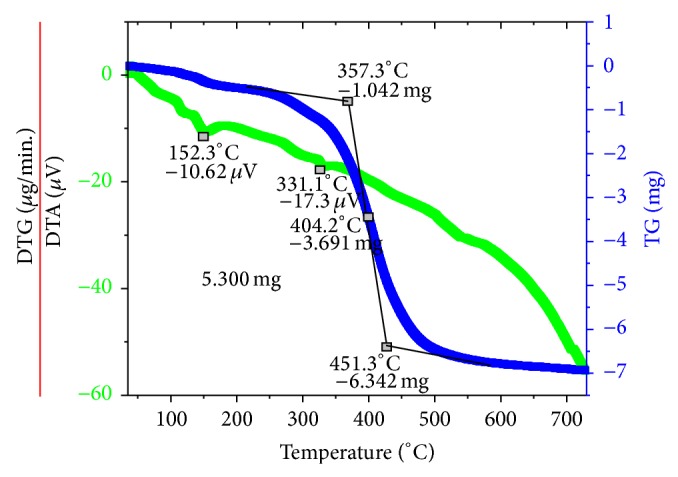
DTA of sample 10.

*Differential Thermal Analysis (DTA)*. The DTA analysis of sample 10 is given in Figure 6. Two endothermic peaks show the melting points of sample 10. The first two endothermic peaks in the range of 100–200°C confirm the removal of unreacted formalin. The second peak shows the melting point of melamine at 331°C. The DTA analysis also confirmed the decomposition of MF resin.

## 4. Conclusion

Melamine formaldehyde resins have successfully been synthesized at different molar ratios of melamine and formaldehyde, temperature, and pH. It was concluded that solid content increases with an increase in temperature. The maximum yield 69.7% was obtained at 75°C temperature. The maximum molecular weight was obtained from sample 10 which is 114218. Finally, the highest residual weight of 14.125% was obtained with sample 10 at 800°C.

## Figures and Tables

**Table 1:** Different molar concentration of melamine and formaldehyde resin at temperature 75°C and pH 8.5.

Number of samples	Molar conc. of melamine	Molar conc. of formaldehyde	% age of solid content
1	0.13 M	3 M	40
2	0.14 M	3 M	43.5
3	0.15 M	3 M	48.7
4	0.16 M	3 M	53.5
5	0.17 M	3 M	57.6
6	0.18 M	3 M	60.2
7	0.19 M	3 M	63.5
8	0.20 M	3 M	65.8
9	0.21 M	3 M	67.4
10	0.22 M	3 M	69.7

**Table 2:** Dependence of the flow time, relative viscosity, specific viscosity, Δ = *η*_sp−In⁡_ *η*_*r*_, and Δ/*c* ^2^ on the concentration of sample 6.

Conc. (g/mL)	Flow time (seconds)	*η*_*r*_	*η*_sp_	Δ = *η*_sp−In⁡_ *η*_*r*_	Δ/c^2^
8 × 10^−4^	128.18	1.077	0.077	0.0030	4688
7 × 10^−4^	127.25	1.069	0.069	0.0019	4042
6 × 10^−4^	125.09	1.051	0.051	0.0012	3333
5 × 10^−4^	124.00	1.0420	0.0420	0.00084	3367
4 × 10^−4^	122.80	1.0319	0.0319	0.00046	2915

**Table 3:** Dependence of the concentration of sample (7–10) on Δ/*c* ^2^.

Conc. g/mL	Sample 7	Sample 8	Sample 9	Sample 10
8 × 10^−4^	6094	8185	8561	9375
7 × 10^−4^	6111	7959	8367	9591
6 × 10^−4^	5833	7777	8333	9444
5 × 10^−4^	5670	7524	8096	9107
4 × 10^−4^	5362	6875	7560	8454

**Table 4:** Dependence of molecular weight of MF samples (6–10) on monomers ratios.

Sample	Monomers ratio	Molecular weight
6	35 : 65	44860
7	36.5 : 63.5	78829
8	38.3 : 61.7	96158
9	41.1 : 58.9	103703
10	42.4 : 58.6	114218

## References

[1] Binder W. H., Dunky M. (2002). Melamine-formaldehyde resins. *Encyclopedia of Polymer Science and Technology*.

[2] Ayrilmis N. (2012). Enhancement of dimensional stability and mechanical properties of light MDF by adding melamine resin impregnated paper waste. *International Journal of Adhesion and Adhesives*.

[3] Bishopp J., Ebnesajjad S. (2011). 13-Adhesives for aerospace structures. *Handbook of Adhesives and Surface Preparation*.

[4] Clad W. (1983). Developments and problems in adhesives used for particle board manufacture. *International Journal of Adhesion and Adhesives*.

[5] Ferra J. M. M., Ohlmeyer M., Mendes A. M., Costa M. R. N., Carvalho L. H., Magalhães F. D. (2011). Evaluation of urea-formaldehyde adhesives performance by recently developed mechanical tests. *International Journal of Adhesion and Adhesives*.

[6] Majda P., Skrodzewicz J. (2009). A modified creep model of epoxy adhesive at ambient temperature. *International Journal of Adhesion and Adhesives*.

[7] Wang J., Jiang N., Jiang H. (2009). Effect of the evolution of phenol-formaldehyde resin on the high-temperature bonding. *International Journal of Adhesion and Adhesives*.

[8] Girods P., Dufour A., Rogaume Y., Rogaume C., Zoulalian A. (2008). Thermal removal of nitrogen species from wood waste containing urea formaldehyde and melamine formaldehyde resins. *Journal of Hazardous Materials*.

[9] Wang S.-Y., Yang T.-H., Lin L.-T., Lin C.-J., Tsai M.-J. (2007). Properties of low-formaldehyde-emission particleboard made from recycled wood-waste chips sprayed with PMDI/PF resin. *Building and Environment*.

[10] Dante R. C., Santamaria D. A., Gil J. M. (2009). Crosslinking and thermal stability of thermosets based on novolak and melamine. *Journal of Applied Polymer Science*.

[11] Naz M. Y., Sulaiman S. A., Ariwahjoedi B., Shaari K. Z. K. (2014). Characterization of modified tapioca starch solutions and their sprays for high temperature coating applications. *The Scientific World Journal*.

[12] Santhanalakshmi J. (1987). Studies on the thermal decomposition of thermosetting aniline-formaldehyde resins. *Thermochimica Acta*.

[13] Stark W. (2010). Investigation of curing behaviour of melamine/phenolic (MP) thermosets. *Polymer Testing*.

[14] Christensen G. (1980). Analysis of functional groups in amino resins. *Progress in Organic Coatings*.

[15] Ricciotti L., Roviello G., Tarallo O. (2013). Synthesis and characterizations of melamine-based epoxy resins. *International Journal of Molecular Sciences*.

[16] Kim S., Kim H.-J. (2006). Thermal stability and viscoelastic properties of MF/PVAc hybrid resins on the adhesion for engineered flooring in under heating system; ONDOL. *Thermochimica Acta*.

[17] Batista M. A. J., Moraes R. P., Barbosa J. C. S., Oliveira P. C., Santos A. M. (2011). Effect of the polyester chemical structure on the stability of polyester-melamine coatings when exposed to accelerated weathering. *Progress in Organic Coatings*.

[18] Bauer D. R. (1986). Melamine/formaldehyde crosslinkers: characterization, network formation and crosslink degradation. *Progress in Organic Coatings*.

[19] Ullah S., Ahmad F., Yusoff P. S. M. M. (2013). Effect of boric acid and melamine on the intumescent fire-retardant coating composition for the fire protection of structural steel substrates. *Journal of Applied Polymer Science*.

[20] Zanetti M., Pizzi A., Beaujean M., Pasch H., Rode K., Dalet P. (2002). Acetals-induced strength increase of melamine-urea-formaldehyde (MUF) polycondensation adhesives. II. Solubility and colloidal state disruption. *Journal of Applied Polymer Science*.

[21] Bal A., Acar I., Güçlü G. (2012). A novel type nanocomposite coating based on alkyd-melamine formaldehyde resin containing modified silica: preparation and film properties. *Journal of Applied Polymer Science*.

[22] Pizzi A., Pizzi A., Mittal K. L. (2002). Melamine-formaldehyde resins. *Handbook of Adhesive Technology*.

[23] Nemli G., Usta M. (2004). Influences of some manufacturing factors on the important quality properties of melamine-impregnated papers. *Building and Environment*.

[24] Fink J. K. (2013). Melamine resins. *Reactive Polymers Fundamentals and Applications*.

[25] Jahromi S. (1999). The storage stability of melamine formaldehyde resin solutions: III. Storage at elevated temperatures. *Polymer*.

[26] Allcock H. R., Lampe F. W. (1980). *Contemporary Polymer Chemistry*.

[27] Pizzi A., Ibeh C. C., Dodiuk H., Goodman S. H. (2014). 4-Aminos. *Handbook of Thermoset Plastics*.

[28] Zanetti M., Pizzi A. (2003). Low addition of melamine salts for improved melamine-urea-formaldehyde adhesive water resistance. *Journal of Applied Polymer Science*.

[29] Tsai P.-F., Kuo W.-L., Shau M.-D. (2013). Thermal properties improvement of bismaleimide resin by a new phosphorus-containing polycyclic bismaleimide. *Journal of the Chinese Chemical Society*.

[30] Devallencourt C., Saiter J. M., Fafet A., Ubrich E. (1995). Thermogravimetry/Fourier transform infrared coupling investigations to study the thermal stability of melamine formaldehyde resin. *Thermochimica Acta*.

[31] Ullah S., Ahmad F. (2014). Effects of zirconium silicate reinforcement on expandable graphite based intumescent fire retardant coating. *Polymer Degradation and Stability*.

